# Automated subfield volumetric analysis of hippocampus in temporal lobe epilepsy using high-resolution T2-weighed MR imaging

**DOI:** 10.1016/j.nicl.2016.06.008

**Published:** 2016-06-13

**Authors:** Daichi Sone, Noriko Sato, Norihide Maikusa, Miho Ota, Kaoru Sumida, Kota Yokoyama, Yukio Kimura, Etsuko Imabayashi, Yutaka Watanabe, Masako Watanabe, Mitsutoshi Okazaki, Teiichi Onuma, Hiroshi Matsuda

**Affiliations:** a Department of Radiology, National Center of Neurology and Psychiatry, 4-1-1 Ogawa-Higashi, Kodaira, Tokyo 187-8551, Japan; b Department of Neuropsychiatry, Graduate School of Medicine, The University of Tokyo, 7-3-1, Hongo, Bunkyo, Tokyo 113-8654, Japan; c Integrative Brain Imaging Center, National Center of Neurology and Psychiatry, 4-1-1 Ogawa-Higashi, Kodaira, Tokyo 187-8551, Japan; d Department of Psychiatry, National Center of Neurology and Psychiatry, 4-1-1 Ogawa-Higashi, Kodaira, Tokyo 187-8551, Japan; e Musashino-Kokubunji Clinic, 4-1-9-3, Honcho, Kokubunji, Tokyo 185-0012, Japan

**Keywords:** TLE, temporal lobe epilepsy, HS, hippocampal sclerosis, ASHS, automatic segmentation of hippocampal subfields, CA, cornu ammonis, DG, dentate gyrus, Temporal lobe epilepsy, Hippocampal subfields, Automatic segmentation, MRI, Hippocampal sclerosis

## Abstract

**Background and purpose:**

Automated subfield volumetry of hippocampus is desirable for use in temporal lobe epilepsy (TLE), but its utility has not been established. Automatic segmentation of hippocampal subfields (ASHS) and the new version of FreeSurfer software (ver.6.0) using high-resolution T2-weighted MR imaging are candidates for this volumetry. The aim of this study was to evaluate hippocampal subfields in TLE patients using ASHS as well as the old and new versions of FreeSurfer.

**Materials and methods:**

We recruited 50 consecutive unilateral TLE patients including 25 with hippocampal sclerosis (TLE-HS) and 25 without obvious etiology (TLE-nonHS). All patients and 45 healthy controls underwent high-resolution T2-weighted and 3D-volume T1-weighted MRI scanning. We analyzed all of their MR images by FreeSurfer ver.5.3, ver.6.0 and ASHS. For each subfield, normalized *z*-scores were calculated and compared among groups.

**Results:**

In TLE-HS groups, ASHS and FreeSurfer ver.6.0 revealed maximal *z*-scores in ipsilateral cornu ammonis (CA) 1, CA4 and dentate gyrus (DG), whereas in FreeSurfer ver.5.3 ipsilateral subiculum showed maximal z-scores. In TLE-nonHS group, there was no significant volume reduction by either ASHS or FreeSurfer.

**Conclusions:**

ASHS and the new version of FreeSurfer may have an advantage in compatibility with existing histopathological knowledge in TLE patients with HS compared to the old version of FreeSurfer (ver.5.3), although further investigations with pathological findings and/or surgical outcomes are desirable.

## Highlights

• Automated hippocampal subfield analysis is needed in temporal lobe epilepsy (TLE).
• Automatic segmentation hippocampal subfields (ASHS) and FreeSurfer are available.
• 50 TLE patients (25 with hippocampal sclerosis (HS)) were analyzed.
• ASHS and new version of FreeSurfer may have an advantage in compatibility.
• High-resolution T2-weighted images might help better automatic segmentation in TLE.

## 1 Introduction

Temporal lobe epilepsy (TLE) is the most common focal epilepsy in adults (Hauser et al., 1996). Hippocampal sclerosis (HS) is considered the most frequent etiology of TLE and shows hippocampal atrophy and T2/FLAIR hyperintensity on magnetic resonance imaging (MRI) (Cendes et al., 2014). TLE with HS is classified as a distinct constellation (Berg et al., 2010) in which surgical treatment can often achieve preferable seizure outcome compared to drug therapy (Wiebe et al., 2001). A recent pathological classification identifies three HS subtypes according to subfields of neural loss (Cendes et al., 2014, Blumcke et al., 2013), and HS subtypes are considered to have different clinical courses and surgical outcomes (Thom, 2014, Na et al., 2015).

Regarding structural neuroimaging of hippocampal subfields, recent studies using manual procedures have achieved some successful results (Mueller et al., 2009, Coras et al., 2014, Na et al., 2014). However, manual segmentation requires extensive training as well as time consuming for its performance (Yushkevich et al., 2010). In addition, the presently recommended MRI protocol in epilepsy is still at 1.5- or 3.0-T in clinical practice (Ramli et al., 2015), although some studies were performed with 4.0- to 7.0-T MRI (Mueller et al., 2009, Coras et al., 2014). An automated procedure using 1.5- or 3.0-T MRI is thus desirable for practical clinical application in TLE.

The authors of a 2014 study attempted to analyze hippocampal subfields automatically using FreeSurfer and T1-weighed images (Schoene-Bake et al., 2014). In TLE, but the results showed insufficient correlation with pathological findings in some subfields. In addition, the validity of hippocampal segmentation by FreeSurfer ver.5.3 faced various criticisms (Wisse et al., 2014). In fact, no clinically useful method has ever been established in this field. Responding to those criticisms, the new version of FreeSurfer (ver.6.0) has been developed for more precise subfields segmentation of hippocampus using high-resolution T2-weighted images (Iglesias et al., 2015). Furthermore, the automatic segmentation of hippocampal subfields (ASHS) has emerged as a useful fully automatic algorithm for multi-atlas-based labeling of hippocampal subfields and adjacent cortical subregions also using high-resolution coronal T2-weighted images (Yushkevich et al., 2010, Yushkevich et al., 2015). T2-weighted images were also suggested to be more suitable for analyses of hippocampus (Wisse et al., 2014). In addition, these automated methods may detect slight abnormalities of hippocampus in TLE cases without clear etiology (so-called “MRI-negative” TLE cases), which were reported by manual segmentation procedures (Kim et al., 2013, Maccotta et al., 2015). The aim of this study was to evaluate hippocampal subfields in TLE with HS and MRI-negative TLE using these new automated methods, and to compare the results with those of the hitherto-existing method. We hypothesized that segmentation methods with high-resolution T2-weighted images would detect more severe atrophy in the supposed subfields such as CA1 or CA4/DG, especially in TLE with HS cases.

## 2 Materials and methods

### 2.1 Participants

We recruited patients with unilateral TLE who were examined at our institute between December 2014 and July 2015. The diagnosis of TLE was based on presence of simple or complex partial seizures consistent with TLE, and focal epileptiform discharge predominantly in unilateral temporal area as observed by conventional scalp electroencephalogram. After diagnosis of TLE, all patients underwent conventional MRI for visual evaluation of epileptogenic lesions by one experienced neuroradiologist (N.S.). Patients with the following criteria were excluded: a significant medical history of acute encephalitis, meningitis, severe head trauma, or ischemic encephalopathy; suspicious epileptogenic lesions (e.g., tumor, cortical dysplasia or vascular malformation) on MRI other than ipsilateral HS at the abnormal electroencephalogram side; or epileptic paroxysms in extra-temporal regions on electroencephalogram.

We divided the eligible patients into two groups based on the existence or non-existence of unilateral HS by the following criteria: ipsilateral reduced hippocampal volume; increased T2 signal on the hippocampus; and abnormal morphology (i.e., a loss of internal architecture of the stratum radiatum, a thin layer of white matter that separates the dentate nucleus and Ammon's horn). Clinical data including gender, age, onset age of epilepsy and number of anti-epileptic drugs were also investigated.

A final total of 50 consecutive unilateral TLE patients were enrolled, of which 25 patients showed HS on MRI (TLE-HS group, 13 females and 12 males, 44.7 ± 11.8 years). No abnormalities were found in the other 25 patients (TLE-nonHS group, 14 females and 11 males, 42.9 ± 16.4 years). We also recruited 45 healthy age-matched subjects (23 females and 22 males, 42.6 ± 16.4 years) as a control group. There were no significant differences among the three groups in age and gender (one-way ANOVA and Pearson's χ^2^ test). TLE-HS group had earlier onset age (16.2 ± 11.9, 29.0 ± 18.6 years, *p* < 0.01 by *t*-test) and took more anti-epileptic drugs (2.48 ± 1.16, 1.60 ± 0.96, *p* < 0.01 by *t*-test) compared to TLE-nonHS group.

All participants gave written informed consent. The study was approved by the Institutional Review Board at the National Center of Neurology and Psychiatry Hospital.

### 2.2 MRI acquisitions

The MRI for all participants was performed on a 3.0-T MR system with a 32-channel coil (Philips Medical Systems, Best, The Netherlands). The parameters of sequences were as follows. Three-dimensional (3D) sagittal T1-weighted magnetization prepared rapid acquisition with gradient echo (MPRAGE) images: repetition time (TR)/echo time (TE): 7.12 ms/3.4 ms; flip angle (FA): 10°; number of excitations (NEX): 1; 0.81 × 0.81 mm^2^ in plane resolution, 0.6 mm effective slice thickness with no gap, 300 slices, matrix of 260 × 320; 26 × 24 cm field of view (FOV); acquisition time 4:01 min. High-resolution T2-weighted images were obtained as follows. TR/TE, 6000/78 ms; FA, 90°; NEX, 2, 0.43 × 0.43 mm^2^ in plane resolution, 2-mm slice thickness with no gap; 32 slices, matrix of 476 × 377, 22 × 24 cm FOV, acquisition time 6:00 min.

We also added a routine MRI examination by the following three protocols. Transverse conventional T1-weighted images: TR/TE: 602/8.0 ms, FA 70°, NEX 1, thickness 3.0 mm with 1.5-mm gap, 34 slices, matrix 256 × 174, 23 × 18 cm FOV, acquisition time 3:33 min. Transverse turbo spin echo T2-weighted images: TR/TE: 4704/80 ms, FA 90°, NEX 2, thickness 3.0 mm with 1.5-mm gap, 34 slices, matrix 368 × 215, 23 × 18 cm FOV, acquisition time 2:49 min. Coronal fluid-attenuated inversion recovery (FLAIR) images: TR/TE 10,000/120 ms, inversion time 2450 ms, FA 120°, NEX 2, thickness 3.0 mm with 1.5-mm gap, 34 slices, matrix 272 × 144, 23 × 18 cm FOV, acquisition time 3:00 min.

All participants underwent the same MR machine scan, and the same images were applied to the automated volumetric processes. In addition, experienced neuroradiologists visually confirmed the good or fair parcellation in each volumetric analysis.

### 2.3 FreeSurfer volumetry of hippocampal subfields

**Fig. 1:**
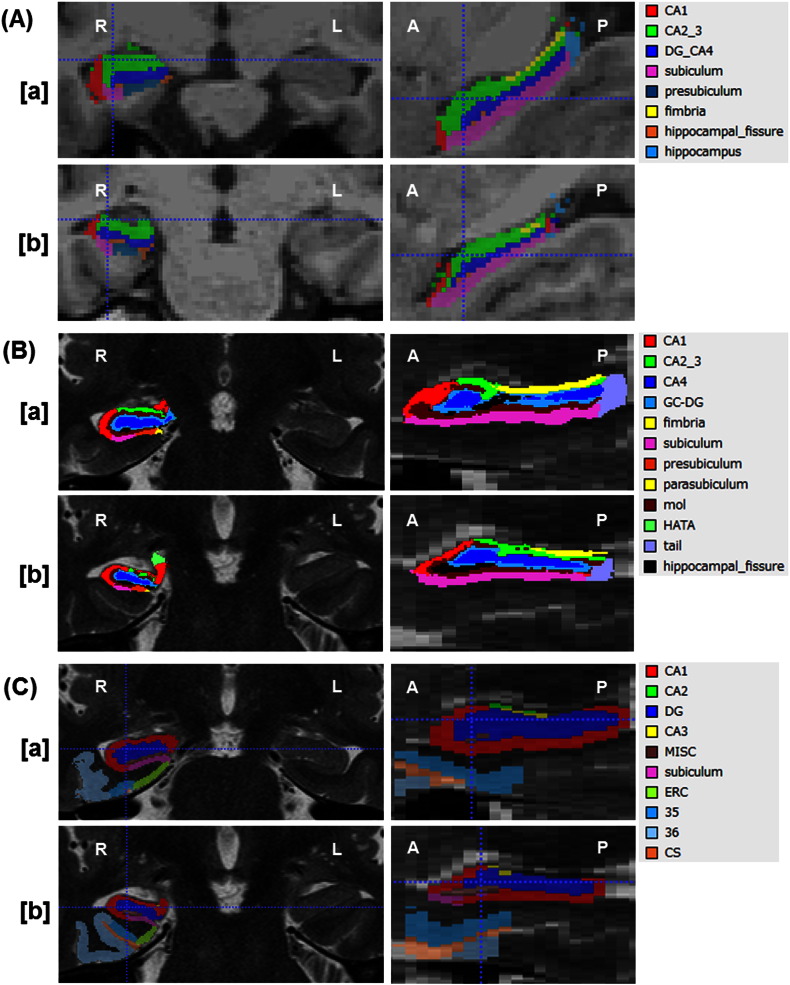
(A): Examples of automatic hippocampal segmentation results by FreeSurfer ver.5.3 on 3D T1-weighed MRI in a healthy subject (a) and a patient with right hippocampal sclerosis (b). (B): Examples of automatic hippocampal segmentation results by FreeSurfer ver.6.0 on high-resolution T2-weighted MRI in the same participants as in (A). (C): Examples of automatic hippocampal segmentation results by ASHS on high-resolution T2-weighted MRI in the same participants as in (A).

FreeSurfer software ver.5.3 was used for calculation of hippocampal subregions volumes using 3D T1-weighted images. This hippocampal subregion segmentation technique based on a prior probabilistic atlas, and the Baysian modeling approach is fully automatic and can be found online (www.freesurfer.net/fswiki/HippocampalSubfieldSegmentation) (Van Leemput et al., 2009). The calculated subregions were cornu ammonis (CA) 1, CA2/3, CA4/dentate gyrus (DG), fimbria, subiculum, presubiculum, hippocampus, and hippocampal fissure (Fig. 1).

FreeSurfer software ver.6.0 (www.freesurfer.net/fswiki/HippocampalSubfields) was also used for evaluation of hippocampal subregions volumes with both 3D T1- and high-resolution T2-weighted images (Iglesias et al., 2015). Officially, FreeSurfer ver.6.0 has not been released yet, but in this study the module corresponds to the latest development version (FreeSurfer6.0 dev-20,150,808) which will be part of the upcoming ver.6.0 of the package. Thirteen regions were calculated including CA1, CA2/3, CA4, granule cell layer of DG, fimbria, subiculum, presubiculum, parasubiculum, molecular layer, hippocampus-amygdala-transition-area, hippocampal tail, whole hippocampus and hippocampal fissure (Fig. 1). Although FreeSurfer ver.6.0 accepts MRI scans acquired with weightings other than T2, we adopted high-resolution T2-weighted images because ASHS needs them and we considered the same images would be appropriate for comparison.

### 2.4 ASHS volumetry of hippocampal subfields

We also applied the same both 3D T1- and high-resolution T2-weighted images obtained from our participants to the open-source ASHS software (https://sites.google.com/site/hipposubfields/) (Yushkevich et al., 2015). After we selected “UPenn PMC Atlas” (Yushkevich et al., 2015) as the Atlas Set, the software calculated the volumes of each subfield fully automatically with a combination of multi-atlas label fusion and learning-based error correction. Ten regions of interest were delineated: CA1, CA2, CA3, DG, subiculum, entorhinal cortex, Brodmann area 35, Brodmann area 36, collateral sulcus and miscellaneous parts (Fig. 1).

### 2.5 Statistical analysis

Analyses were performed using SPSS software (ver. 22.0 Japan, Tokyo). For our comparisons of each subfield volumes among the TLE-HS, TLE-nonHS, and control groups, we performed analysis of covariance (ANCOVA) using age, gender and intracranial volume (ICV), which was calculated by each targeted software of the analysis, as nuisance covariates, plus post-hoc Bonferroni correction. A *p*-value < 0.05 was considered significant.

Using *z*-scores, we aimed to compare the detectability of subfields' atrophy among the different segmentation methods. In each volumetric technique and each subfield, we calculated volume *z*-scores by following formula: volume *z*-score = (volume_subject_ – mean volume_control_) / standard deviation volume_control_.

## 3 Results

### 3.1 Volumetric analyses of hippocampal subfields for TLE-HS group

Table 1, Table 2, Table 3 present hippocampal subfield volumes calculated by FreeSurfer ver.5.3, ver.6.0 and ASHS, respectively. For both sides of focus, significant mean volume reductions were found only in the ipsilateral hemisphere compared to the control group. Beyond that, a weak expansion of hippocampal fissure in right TLE patients was found only by FreeSurfer ver.5.3 (Table 1).

**Fig. 2:**
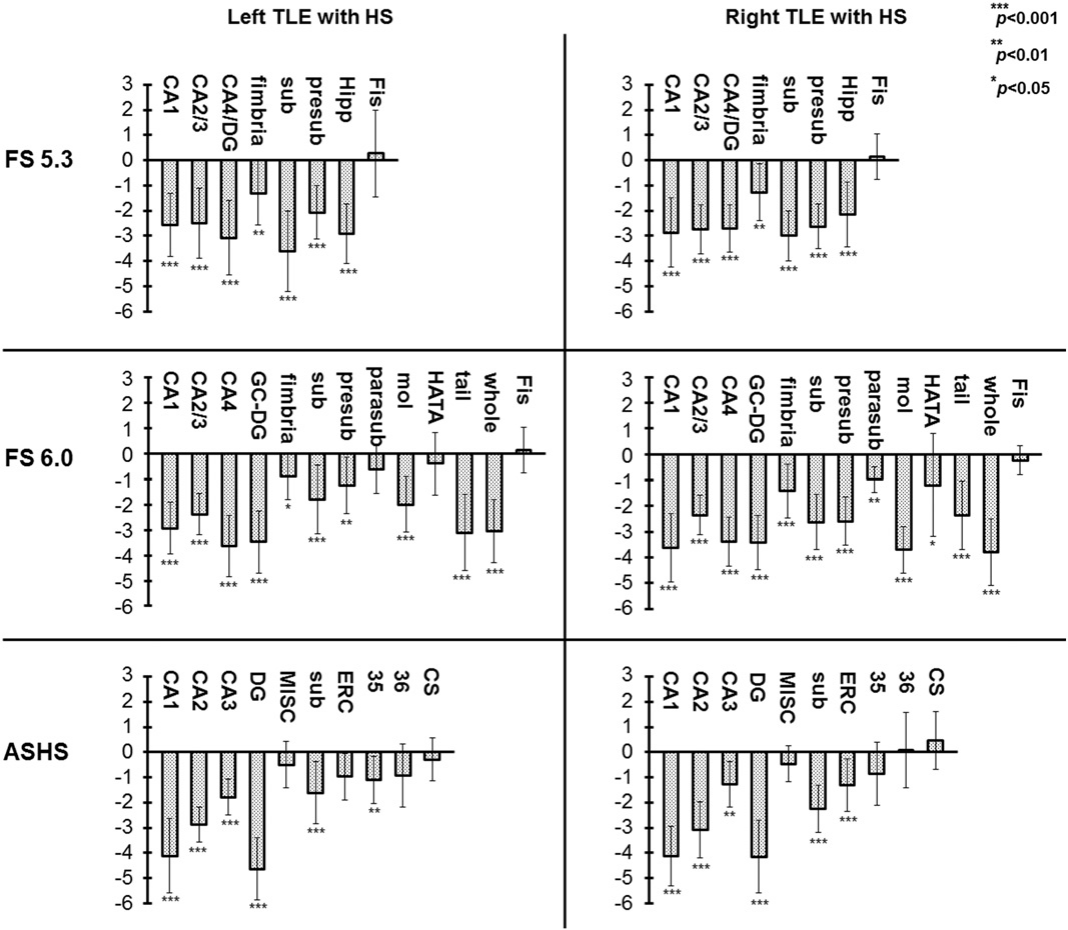
Mean (± SD) z-scores of the ipsilateral hippocampal subfields calculated by FreeSurfer (FS) ver.5.3, ver.6.0 and ASHS in TLE with HS. *p-values of the mean volumes compared to the control group by Bonferroni correction following an ANCOVA. sub: subiculum, presub: presubiculum, Hipp: hippocampus, Fis: hippocampal fissure, GC-DG: granule cell layer of DG, parasub: parasubiculum, mol: molecular layer, HATA: hippocampus-amygdala-transition-area, tail: hippocampal tail, whole: whole hippocampus, MISC: miscellaneous parts, ERC: entorhinal cortex, 35/36: Brodmann area 35/36, CS: collateral sulcus.

Mean *z*-scores in the ipsilateral side are shown in Fig. 2, which allows us to evaluate the selectivity of atrophy in each subfield by each segmentation method. In FreeSurfer ver.5.3, the maximal *z*-scores were observed in ipsilateral subiculum and most of ipsilateral subfields showed similar values. On the other hand, the ipsilateral CA1, CA4 or DG showed the maximal *z*-scores in FreeSurfer ver.6.0 or ASHS.

### 3.2 Volumetric analyses of hippocampal subfields for TLE-nonHS group

Table 1, Table 2, Table 3 also show subfield volumes of TLE-nonHS group. FreeSurfer ver.6.0 detected significant volume increases in left hippocampus-amygdala-transition-area in both left and right TLE (mean ± SD *z*-scores: 1.59 ± 1.58, 1.01 ± 1.37, respectively). There would be similar tendency in the right hippocampus-amygdala-transition-area, but that's not significant. No other significant volume reductions or z-scores were found by all the segmentation methods.

## 4 Discussion

The present study provided hippocampal subfield volumes on MRI in patients with unilateral TLE with or without HS and healthy subjects, calculated automatically by the new and old versions of FreeSurfer and ASHS. To our best knowledge, this is the first study to apply ASHS and FreeSurfer ver.6.0 for evaluation of the hippocampus in patients with TLE. Our results showed that ASHS and FreeSurfer ver.6.0 detected severe volume loss of ipsilateral CA1 and CA4/DG in TLE patients with HS compared with the other subfields and compared with the old version of FreeSurfer, which accords with pathological findings of HS (Thom, 2014). The two methods with high-resolution T2-weighted images may thus become clinically useful to evaluate detailed hippocampal subfields in TLE patients.

In the present study, we obtained inconsistent volume estimation of FreeSurfer ver.5.3 findings with anatomical evidence of hippocampus. For example, FreeSurfer ver.5.3 estimated CA2/3 as the largest subfield, whereas CA1 was the smallest in healthy subjects (Table 1) despite the existence of anatomical information that CA1 is the largest and CA2/3 is the smallest subfield (Simic et al., 1997). On the other hand, ASHS and FreeSurfer ver.6.0 commonly estimated CA1 as the largest part (Table 2, Table 3). But ASHS produced larger volumes for CA1 than FreeSurfer, and smaller volumes for CA2–3. Histology studies have reported volumes around 600–700 mm^3^ for CA1 and 100–200 mm^3^ for CA2–3 (Simic et al., 1997), and then ASHS may have a tendency to underestimate CA2–3. In addition, severe ipsilateral CA2 volume loss in ASHS could be inconsistent with the pathology, because CA2 should be relatively spared in HS (Thom, 2014). Probably, ASHS may need an improvement in this point. In TLE-HS group, the results of ASHS and FreeSurfer ver.6.0 showed severely low z-scores in ipsilateral CA1 and CA4/DG, whereas FreeSurfer ver.5.3 captured the most severe volume reductions in ipsilateral subiculum, although the main pathological lesions of HS should be those of CA and DG (Cendes et al., 2014, Thom, 2014). The reason for this result might be because large parts of the subfields are assigned to adjacent subfields in FreeSurfer ver.5.3, as was reported (Wisse et al., 2014). The two other methods' results — i.e., in the unilateral TLE patients with HS ipsilateral CA1 and CA4/DG showed more severe volume reductions compared to other subfields — accords with the pathological findings of HS (Thom, 2014). For clinical practice, the running time of the software would be important. The average time for the analysis of one subject was about 50 min with Q-option and Sun. Grid Engine in ASHS (26 h without Q-option), whereas FreeSurfer ver.6.0 required about 18 h per person on average (16 h on the main recon-all pipeline, 2 h on the subfield segmentation), using our computer (Gird Computing Server, CPU Intel Xeon × 5690 (3.46 GHz, 6 cores, 12 MB L3Cache)*2 cpu, Memory 48 GB).

In 2013, International League Against Epilepsy (ILAE) classified HS into three groups based on histological patterns of subfield neuronal loss and gliosis (Blumcke et al., 2013). HS type 1 is the most common (60%–80%) and shows neuronal loss in CA1, CA3 and CA4/DG with relative sparing of CA2 (Thom, 2014). Types 2 and 3 are relatively rare (< 10%). Type 2 shows complete neuronal loss in CA1 with rather mild pathology in all other subfields, whereas Type 3 presents predominant neuronal loss in CA4/DG (Cendes et al., 2014). Although TLE is considered surgically treatable in general, a certain number of patients experience seizure recurrence within several years (McIntosh et al., 2004). Additionally, in TLE with HS surgical outcome could depend on the three pathological types; types 2 and 3 have poorer surgical outcomes (Na et al., 2015). Another study suggested that DG pathology could be associated with postsurgical seizure outcome and other clinical characteristics (Blumcke et al., 2009). It is thus desirable that the type of HS could be predicted preoperatively because it could lead to a better selection of operative methods as well as prognostic prediction. CA4/DG volumetry on 3.0-T MRI with manual procedure was indicated to have probable prognostic value (Na et al., 2014). The previous attempt to analyze hippocampal subfields of TLE with HS automatically using the old version of FreeSurfer failed to show concordance with pathological neuronal loss in CA2/3 and CA4/DG (Schoene-Bake et al., 2014), and thus more accurate analyses of MRI findings are needed for better evaluations of postsurgical prognoses. Here we applied ASHS and the new version of FreeSurfer to TLE patients with HS for the first time, and our results demonstrated the ability to detect pathologically-concordant atrophy in hippocampal subfields. Although we did not investigate the present patient series' surgical seizure outcomes, we speculate that they could become clinically useful and fully reproducible methods for noninvasive evaluations of postsurgical prognosis as well as pathological types in TLE patients with HS.

We did not observe any significant reductions in mean volumes of hippocampal subfields in TLE-nonHS group, which is without obvious etiology (so-called “MRI-negative” TLE). Although some cases might show low z-scores in some subfields individually, the pathological meaning of such results were not confirmed in this study. As for volumetric analyses of the mesial temporal structure, some earlier studies reported no significant abnormalities in MRI-negative TLE (Mueller et al., 2006, Alhusaini et al., 2013). On the other hand, FreeSurfer ver.6.0 captured volume increases in hippocampus-amygdala-transition-area in TLE-nonHS group. In recent years, an increasing number of cases of TLE showing ipsilateral amygdala enlargement without any other etiologies have been reported (Bower et al., 2003, Lv et al., 2014) and contralateral amygdala enlargement was also suggested (Coan et al., 2013). Possibly, FreeSurfer ver.6.0 might detect such volume increases, although we excluded cases with obvious lesions on MRI. In any case, it appears that we may need a more homogeneous patient population or a greater number of patients to detect volume changes in MRI-negative TLE, and there is much to do in this field.

We also calculated volumes of extra-hippocampal related regions such as entorhinal cortex, Brodmann area 35 and 36 by ASHS. These interesting cortical regions have been indicated to be associated with hippocampus and with memory function (Ranganath and Ritchey, 2012, Guedj et al., 2010). There have been several reports about entorhinal cortex volume reductions in TLE (Bernasconi et al., 2003a, Jutila et al., 2001) and indications of an association with temporal lobe damage (Bernasconi et al., 2003a, Bernasconi et al., 2003b). A study of functional aspects suggested a correlation between entorhinal cortex and memory impairment in TLE (Schwarcz and Witter, 2002). Interestingly, an essential role of generation of ictal discharges or epileptogenicity in entorhinal cortex was described (Ren et al., 2014, Bartolomei et al., 2005). In our study, ASHS also detected significant volume reductions of ipsilateral entorhinal cortex subfields in right TLE-HS group. In ASHS, Brodmann area 35 and 36 account for perirhinal cortex (Yushkevich et al., 2015). Concerning the function of perirhinal cortex in TLE, a relationship between perirhinal cortex and anxiety and recognition memory was suggested in rats (Hannesson et al., 2005). A similar relation between entorhinal/perirhinal cortex and recognition memory was also reported in human beings in a study using ^18^F-FDG-PET (Guedj et al., 2010). Our present findings described significant mean volume reductions in ipsilateral Brodmann area 35 of patients with left TLE with HS, which could possibly be associated with their memory functions. Although further investigations assessing cognitive functions are needed, our results are partly consistent with these previous suggestions about the entorhinal/perirhinal cortex, and the use of ASHS could enable noninvasive evaluations of correlation between entorhinal/perirhinal cortex volumes and cognitive functions or psychiatric symptoms of TLE patients.

This study has several limitations. First, patients' pathological findings and surgical outcomes were not available. For comparison of segmentation methods, a direct comparison with pathological findings, for example using area under receiver operating characteristic curves, must be desirable. Although further investigations that include such data are absolutely needed, the validity and reliability of ASHS have already been demonstrated in healthy subjects and patients with mild cognitive impairment (Yushkevich et al., 2015), and the same goes for FreeSurfer ver.6.0 (Iglesias et al., 2015). Regarding FreeSurfer ver.5.3, a comparison between MRI subfield volumetry and histopathological findings in TLE patients with HS was reported in which there was a significant correlation in CA1 and no significant correlations in CA2/3 or CA4/DG (Schoene-Bake et al., 2014). Since the existing procedure has several problems and new methods are desirable in this field, we propose that it would be meaningful to compare FreeSurfer and ASHS in the same patients with TLE. Second, each subgroup based on existence of HS and laterality of focus had a relatively small number of patients for comparison. However, we were nevertheless able to detect significant differences in various analyses of the TLE-HS group, whereas no significance was found in the TLE-nonHS group except for slight volume increase in hippocampus-amygdala-transition-area. Concerning TLE-nonHS, it would also be difficult to recruit a homogeneous series compared with TLE-HS patients, whose imaging findings are already established. Therefore, our findings about TLE-nonHS should probably be regarded as preliminary. Additionally, there would be a possibility that the altered T2 signal and unsharpness of the dark layer in HS could affect the results of ASHS and FreeSurfer ver.6.0. Regarding this dark layer issue, that would be independent of whether we use FreeSurfer, ASHS or manual segmentation. Especially, CA1 and CA4/DG can be influenced by this problem, because the dark layer separates these subfields. Our visual confirmation of parcellation can also cause a limitation, particularly in FreeSurfer ver.6.0 with detailed small segmentations. Although our results should be interpreted with these limitations, we consider that the two methods with high-resolution T2-weighed images have made some progress compared to the one with only T1-weighed images, which detected more severe atrophy in the subiculum in cases with HS.

## 5 Conclusions

ASHS and FreeSurfer ver.6.0 may have an advantage in compatibility with existing histopathological knowledge compared to FreeSurfer ver.5.3, and may become effective methods to evaluate detailed hippocampal subfields as well as adjacent related cortices in TLE patients, especially those with HS, non-invasively and fully automatically. The use of these automated hippocampal segmentation with high-resolution T2-weighted images may lead to better prognostic predictions and selections of treatment, and a fuller understanding of TLE.

## Conflicts of interest

None.

## Figures and Tables

**Table 1:**
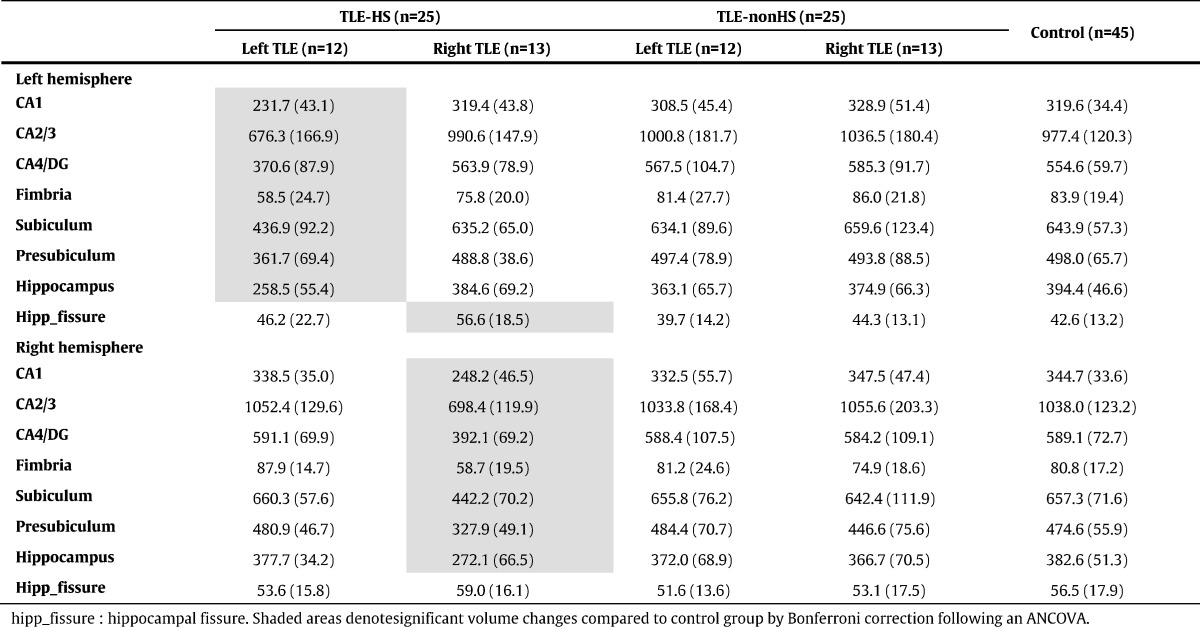
Mean (SD) volumes (mm^3^) of hippocampal subfields calculated by FreeSurfer ver.5.3.

**Table 2:**
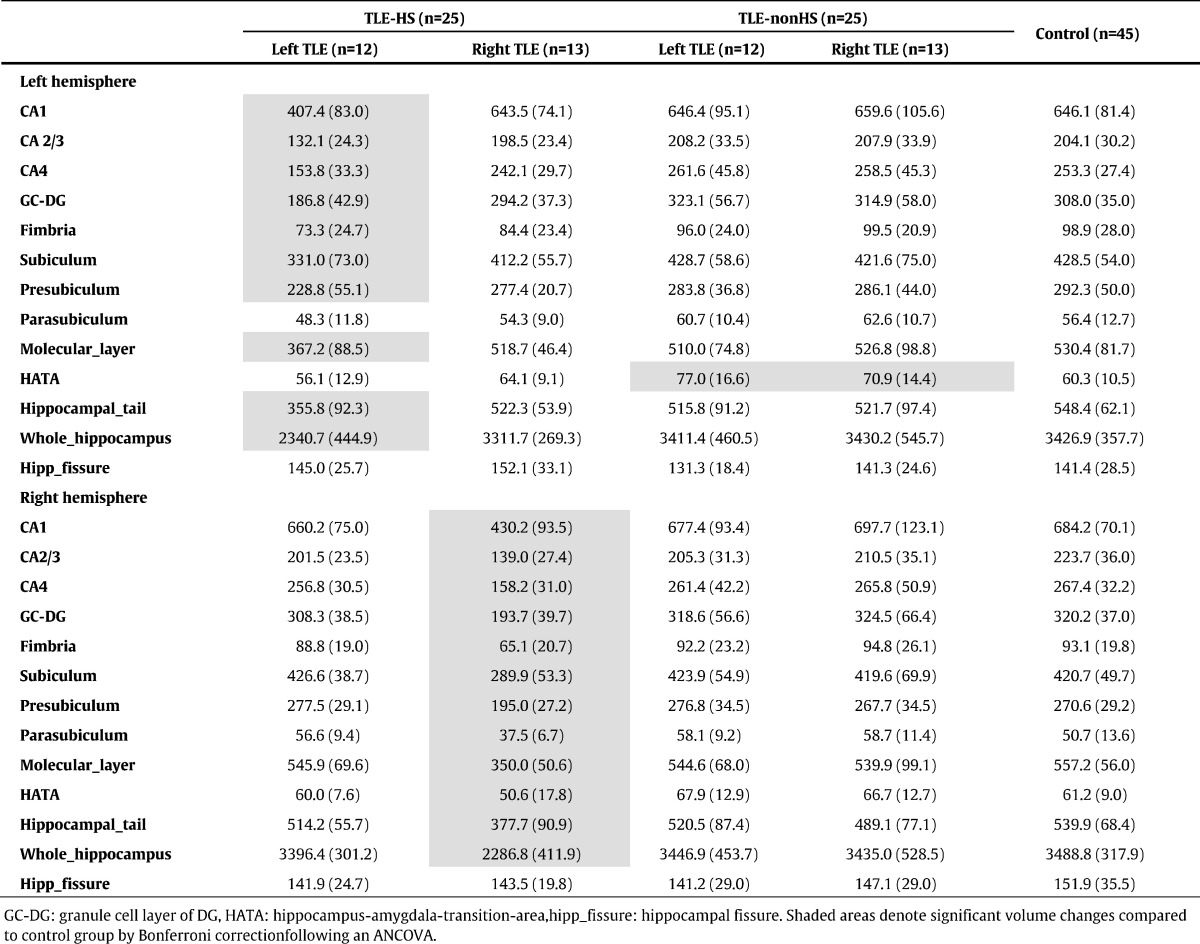
Mean (SD) volumes (mm^3^) of hippocampal subfields calculated by FreeSurfer 6.0.

**Table 3:**
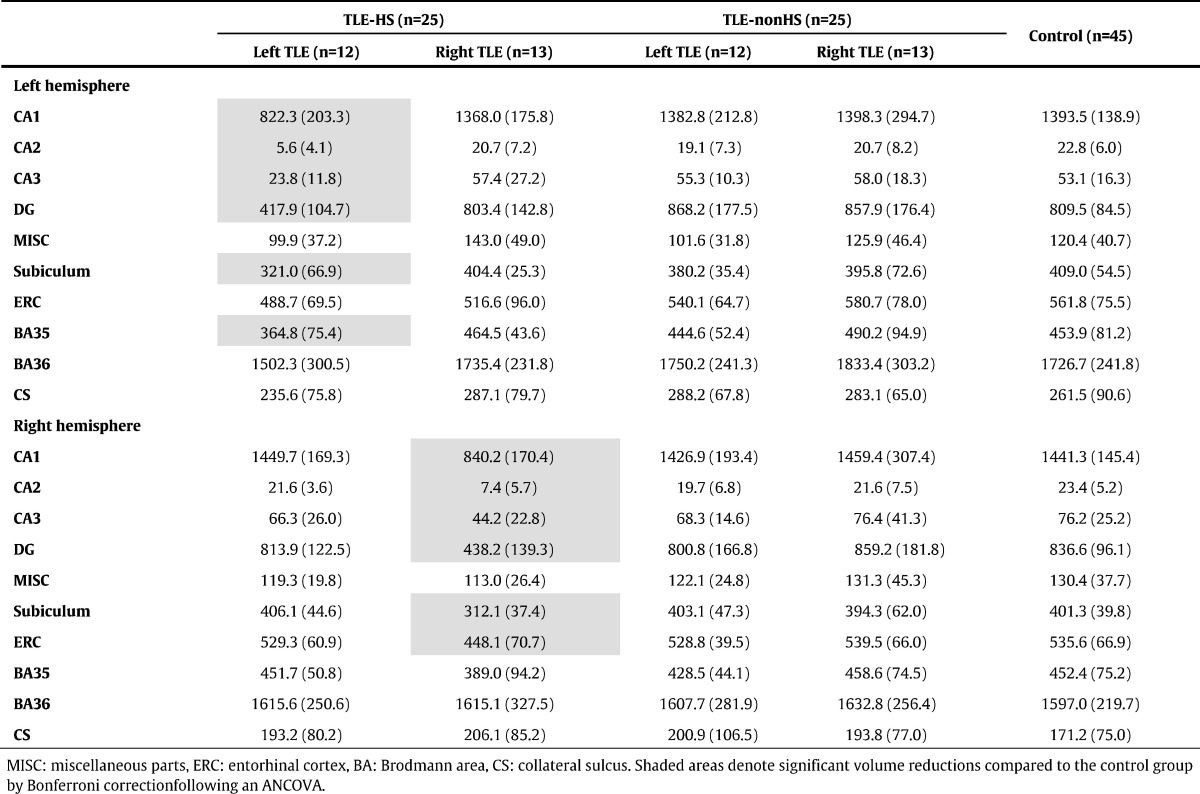
Mean (SD) volumes (mm^3^) of hippocampal subfields calculated by ASHS.
